# *catena*-Poly[[[diaqua­cadmium]-μ-2,2′-(1,2-phenyl­enedi­oxy)diacetato] mono­hydrate]

**DOI:** 10.1107/S1600536811004867

**Published:** 2011-02-16

**Authors:** Huan-Fu Hou, Xiu-Ling Zhang

**Affiliations:** a College of Chemical Engineering, Qingdao University of Science & Technology, Qingdao, Shandong 266042, People’s Republic of China; b Department of Chemistry, Dezhou University, Dezhou, Shandong 253023, People’s Republic of China

## Abstract

In the title coordination complex, {[Cd(C_10_H_8_O_6_)(H_2_O)_2_]·H_2_O}_*n*_ the Cd^II^ atom is seven-coordinated in a distorted penta­gonal–bipyramidal geometry, the penta­gonal plane comprising four O-atom donors from the 2,2′-(1,2-phenyl­enedi­oxy)diacetate chelate ligand together with a bridging carboxyl­ate O-atom donor, with the axial sites occupied by two water mol­ecules. The resulting helical chains extend along the *b* axis and are inter­connected by extensive O—H⋯O hydrogen-bonding inter­actions, which also involve the water mol­ecule of solvation, giving a three-dimensional structure.

## Related literature

For rigid polycarboxyl­ate ligands, see: Liu *et al.* (2010 ▶); Rao *et al.* (2004 ▶). For flexible carboxyl­ate complexes, see: Dai *et al.* (2009 ▶)
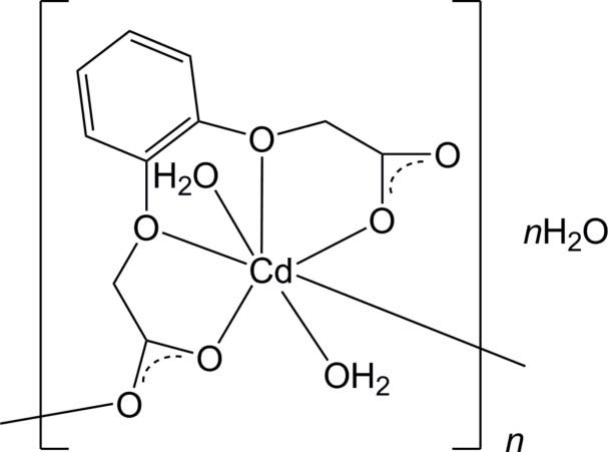


## Experimental

### 

#### Crystal data

[Cd(C_10_H_8_O_6_)(H_2_O)_2_]·H_2_O
*M* *_r_* = 390.61
Monoclinic, 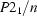

*a* = 7.624 (1) Å
*b* = 7.156 (1) Å
*c* = 23.190 (2) Å
β = 93.083 (1)°
*V* = 1263.4 (3) Å^3^
*Z* = 4
Mo *K*α radiation
μ = 1.77 mm^−1^
*T* = 296 K
0.25 × 0.20 × 0.14 mm

#### Data collection

Bruker APEXII CCD area-detector diffractometer
Absorption correction: multi-scan (*SADABS*; Bruker, 2001 ▶) *T*_min_ = 0.671, *T*_max_ = 0.787
7467 measured reflections
2893 independent reflections
2676 reflections with *I* > 2σ(*I*)
*R*_int_ = 0.017

#### Refinement

*R*[*F* ^2^ > 2σ(*F* ^2^)] = 0.020
*wR*(*F* ^2^) = 0.049
*S* = 1.05
2893 reflections
181 parameters
H-atom parameters constrained
Δρ_max_ = 0.52 e Å^−3^
Δρ_min_ = −0.43 e Å^−3^

### 

Data collection: *APEX2* (Bruker, 2007 ▶); cell refinement: *SAINT* (Bruker, 2007 ▶); data reduction: *SAINT*; program(s) used to solve structure: *SIR97* (Altomare *et al.*, 1999 ▶); program(s) used to refine structure: *SHELXL97* (Sheldrick, 2008 ▶); molecular graphics: *SHELXTL* (Sheldrick, 2008 ▶); software used to prepare material for publication: *WinGX* (Farrugia, 1999 ▶).

## Supplementary Material

Crystal structure: contains datablocks global, I. DOI: 10.1107/S1600536811004867/zs2094sup1.cif

Structure factors: contains datablocks I. DOI: 10.1107/S1600536811004867/zs2094Isup2.hkl

Additional supplementary materials:  crystallographic information; 3D view; checkCIF report

## Figures and Tables

**Table 1:** Hydrogen-bond geometry (Å, °)

*D*—H⋯*A*	*D*—H	H⋯*A*	*D*⋯*A*	*D*—H⋯*A*
O1*W*—H11*W*⋯O3*W*^i^	0.84	2.11	2.892 (3)	154
O1*W*—H12*W*⋯O1^ii^	0.84	1.87	2.686 (2)	164
O2*W*—H21*W*⋯O6^iii^	0.84	2.06	2.873 (3)	165
O2*W*—H22*W*⋯O3*W*^iv^	0.84	2.03	2.860 (3)	170
O3*W*—H31*W*⋯O6	0.84	2.09	2.887 (3)	157
O3*W*—H32*W*⋯O2^v^	0.85	1.99	2.835 (2)	176

Symmetry codes: (i) 
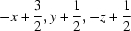
; (ii) 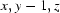; (iii) 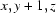; (iv) 
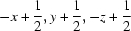
; (v) 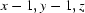.

**Fig. 1.**
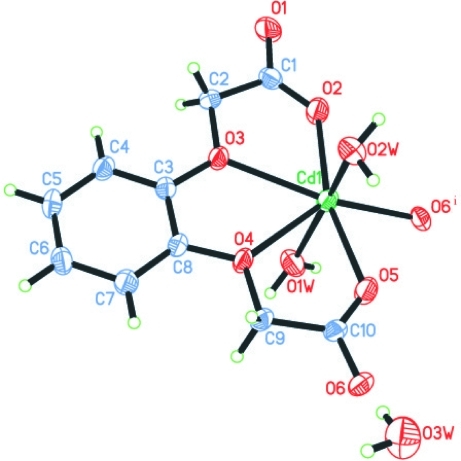
A view of the CdIIcoordination environment of (I) with the atom- labelling scheme. Displacement ellipsoids are drawn at the 50% probability level. H atoms are represented by circles of arbitrary size. For symmetry code (i), see Table 1.

**Fig. 2.**
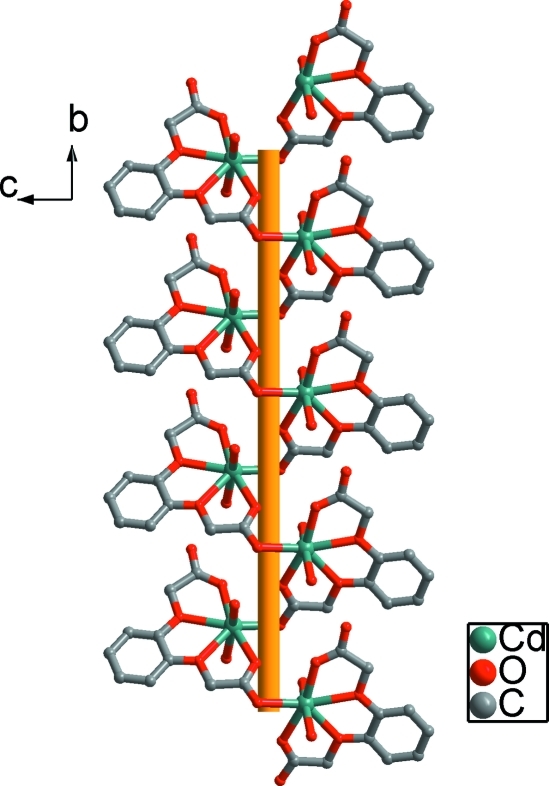
The one-dimensional helical chain structure of (I) viewed along the a axis.

**Fig. 3.**
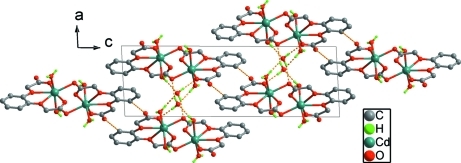
The packing diagram of (I) viewed along the b axis.

## supplementary crystallographic information

### Comment

Rigid polycarboxylate ligands have been employed extensively for the construction of metal-organic polymers, e.g. 1,3-benzenedicarboxylate, 1,3,5-benzenetricarboxylate and 4,4'-biphenyldicarboxylate (Liu *et al.*, 2010; Rao *et al.*, 2004). Compared to rigid ligands with a single conformation, flexible ligands may adopt variable conformations when coordinated to metal ions, making it more difficult to predict and control the final coordination networks. Therefore using flexible ligands in the formation of coordination polymers may generate novel complexes with interesting topologies and attractive properties (Dai *et al.*, 2009). The title compound {[(C_10_H_8_O_6_)(H_2_O)_2_Cd] . H_2_O}_n_ (I), was prepared from the reaction of the flexible carboxylate ligand, the 1,2-phenylenedioxydiacetate dianion (PDA) with Cd^II^ and the structure is reported here.

In (I) (Fig. 1) the Cd^II^ cation is seven-coordinated, involving two carboxyl and two phenoxy O donors (O2, O3, O4, O5) from a PDA ligand [Cd—O range 2.2424 (19) Å–2.5285 (16) Å], and a bridging carboxylate O donor (O6) [Cd—O^i^, 2.3596 (15) Å] [for symmetry code (i), see Table 1], which lie in the pentagonal plane of a distorted pentagonal bipyramid. Two water molecules (O1W, O2W) occupy the axial sites (Cd—O, 2.296 (2), 2.316 (2) Å]. The bond angles about Cd^II^ are in the range of 61.11 (5) to 165.45 (5) °. The mononuclear units of (I) are connected *via* the bridging O6^i^ atoms to give helical chains extending along the *b* axis of the unit cell (Fig. 2). The chains are further inter-connected by extensive hydrogen-bonding interactions (Table 1) involving also the water molecule of solvation (O3W), giving rise to the three-dimensional molecular architecture (Fig. 3).

### Experimental

A mixture of 1,2-phenylenedioxydiacetic acid (H_2_PDA) (0.023 g, 0.1 mmol) and Cd(NO_3_)_2_ . 4H_2_O (0.038 g, 0.1 mmol) in H_2_O (7.0 ml) was placed in a 16 ml Teflon-lined stainless steel vessel and heated to 160 °C for 72 h, giving colorless block crystals of (I), which were collected by filtration. The crystals obtained were washed with water and dried in air. Yield: 0.029 g (74% based on Cd).

### Refinement

All H atoms bonded to C atoms were added according to theoretical models, assigned isotropic displacement parameters and allowed to ride on their respective parent atoms [C—H = 0.93–0.97%A and *U*_iso_(H) = 1.2*U*_eq_(C)]. The H atoms of the water molecules were located from the Fourier map with the O—H distances being fixed at 0.85%A and allowed to ride on their parent oxygen atoms in the final cycles of refinement, with *U*_iso_(H) = 1.2*U*_eq_(O).

### Crystal data

**Table:**

[Cd(C_10_H_8_O_6_)(H_2_O)_2_]·H_2_O	*F*(000) = 776
*M**_r_* = 390.61	*D*_x_ = 2.054 Mg m^−^^3^
Monoclinic, *P*2_1_/*n*	Mo *K*α radiation, λ = 0.71073 Å
Hall symbol: -P 2yn	Cell parameters from 4670 reflections
*a* = 7.624 (1) Å	θ = 2.8–27.5°
*b* = 7.156 (1) Å	µ = 1.77 mm^−^^1^
*c* = 23.190 (2) Å	*T* = 296 K
β = 93.083 (1)°	Block, colorless
*V* = 1263.4 (3) Å^3^	0.25 × 0.20 × 0.14 mm
*Z* = 4	

### Data collection

**Table:**

Bruker APEXII CCD area-detector diffractometer	2893 independent reflections
Radiation source: fine-focus sealed tube	2676 reflections with *I* > 2σ(*I*)
graphite	*R*_int_ = 0.017
φ and ω scans	θ_max_ = 27.6°, θ_min_ = 1.8°
Absorption correction: multi-scan (*SADABS*; Bruker, 2001)	*h* = −9→9
*T*_min_ = 0.671, *T*_max_ = 0.787	*k* = −9→5
7467 measured reflections	*l* = −29→30

### Refinement

**Table:**

Refinement on *F*^2^	Primary atom site location: structure-invariant direct methods
Least-squares matrix: full	Secondary atom site location: difference Fourier map
*R*[*F*^2^ > 2σ(*F*^2^)] = 0.020	H-atom parameters constrained
*wR*(*F*^2^) = 0.049	*w* = 1/[σ^2^(*F*_o_^2^) + (0.0217*P*)^2^ + 0.8227*P*] where *P* = (*F*_o_^2^ + 2*F*_c_^2^)/3
*S* = 1.05	(Δ/σ)_max_ = 0.002
2893 reflections	Δρ_max_ = 0.52 e Å^−^^3^
181 parameters	Δρ_min_ = −0.43 e Å^−^^3^
0 restraints	

### Special details

**Table:**

Geometry. All e.s.d.'s (except the e.s.d. in the dihedral angle between two l.s. planes) are estimated using the full covariance matrix. The cell e.s.d.'s are taken into account individually in the estimation of e.s.d.'s in distances, angles and torsion angles; correlations between e.s.d.'s in cell parameters are only used when they are defined by crystal symmetry. An approximate (isotropic) treatment of cell e.s.d.'s is used for estimating e.s.d.'s involving l.s. planes.
Refinement. Refinement of *F*^2^ against ALL reflections. The weighted *R*-factor *wR* and goodness of fit *S* are based on *F*^2^, conventional *R*-factors *R* are based on *F*, with *F* set to zero for negative *F*^2^. The threshold expression of *F*^2^ > σ(*F*^2^) is used only for calculating *R*-factors(gt) *etc*. and is not relevant to the choice of reflections for refinement. *R*-factors based on *F*^2^ are statistically about twice as large as those based on *F*, and *R*- factors based on ALL data will be even larger.

### Fractional atomic coordinates and isotropic or equivalent isotropic displacement parameters (Å^2^)

**Table:**

	*x*	*y*	*z*	*U*_iso_*/*U*_eq_	
C1	0.9769 (3)	1.4246 (3)	0.10705 (9)	0.0281 (4)	
C2	0.9052 (3)	1.3164 (3)	0.05451 (8)	0.0262 (4)	
H2A	0.8158	1.3898	0.0336	0.031*	
H2B	0.9990	1.2920	0.0289	0.031*	
C3	0.7673 (2)	1.0222 (3)	0.03073 (8)	0.0220 (4)	
C4	0.7833 (3)	1.0442 (3)	−0.02803 (8)	0.0258 (4)	
H4	0.8419	1.1472	−0.0421	0.031*	
C5	0.7109 (3)	0.9109 (3)	−0.06585 (9)	0.0297 (4)	
H5	0.7212	0.9252	−0.1054	0.036*	
C6	0.6246 (3)	0.7584 (3)	−0.04544 (9)	0.0302 (4)	
H6	0.5769	0.6701	−0.0712	0.036*	
C7	0.6081 (3)	0.7354 (3)	0.01373 (9)	0.0281 (4)	
H7	0.5491	0.6324	0.0276	0.034*	
C8	0.6802 (3)	0.8668 (3)	0.05152 (8)	0.0224 (4)	
C9	0.5795 (3)	0.7102 (3)	0.13513 (8)	0.0267 (4)	
H9A	0.6249	0.5924	0.1215	0.032*	
H9B	0.4555	0.7172	0.1236	0.032*	
C10	0.6045 (2)	0.7211 (3)	0.20018 (8)	0.0218 (4)	
Cd1	0.834230 (19)	1.08605 (2)	0.178353 (6)	0.02552 (6)	
O1	1.0404 (3)	1.5791 (2)	0.09652 (8)	0.0474 (5)	
O2	0.9688 (2)	1.3536 (2)	0.15657 (6)	0.0364 (4)	
O3	0.8318 (2)	1.1441 (2)	0.07256 (6)	0.0286 (3)	
O4	0.6707 (2)	0.8613 (2)	0.11069 (6)	0.0296 (3)	
O5	0.6948 (2)	0.8467 (2)	0.22331 (6)	0.0315 (3)	
O6	0.53106 (19)	0.59313 (18)	0.22765 (6)	0.0274 (3)	
O1W	1.0606 (2)	0.8817 (2)	0.16508 (7)	0.0370 (4)	
H11W	1.1442	0.8811	0.1902	0.044*	
H12W	1.0565	0.7756	0.1495	0.044*	
O2W	0.5593 (2)	1.2223 (2)	0.18164 (7)	0.0349 (3)	
H21W	0.5675	1.3249	0.1990	0.042*	
H22W	0.5013	1.1503	0.2021	0.042*	
O3W	0.1714 (2)	0.5083 (3)	0.25089 (8)	0.0464 (4)	
H31W	0.2619	0.5523	0.2366	0.056*	
H32W	0.1072	0.4657	0.2230	0.056*	

### Atomic displacement parameters (Å^2^)

**Table:**

	*U*^11^	*U*^22^	*U*^33^	*U*^12^	*U*^13^	*U*^23^
C1	0.0339 (11)	0.0221 (10)	0.0289 (10)	−0.0027 (8)	0.0077 (8)	−0.0028 (8)
C2	0.0357 (11)	0.0191 (9)	0.0239 (9)	−0.0037 (8)	0.0043 (8)	0.0035 (7)
C3	0.0235 (9)	0.0226 (9)	0.0198 (9)	−0.0007 (7)	0.0004 (7)	−0.0008 (7)
C4	0.0292 (10)	0.0281 (10)	0.0205 (9)	−0.0020 (8)	0.0037 (7)	0.0030 (8)
C5	0.0344 (11)	0.0387 (12)	0.0164 (9)	0.0008 (9)	0.0029 (8)	−0.0009 (8)
C6	0.0362 (11)	0.0331 (11)	0.0211 (9)	−0.0040 (9)	−0.0010 (8)	−0.0056 (8)
C7	0.0321 (10)	0.0279 (10)	0.0243 (9)	−0.0067 (8)	0.0005 (8)	0.0002 (8)
C8	0.0264 (10)	0.0249 (9)	0.0160 (8)	−0.0004 (8)	0.0007 (7)	0.0019 (7)
C9	0.0346 (11)	0.0245 (10)	0.0211 (9)	−0.0092 (8)	0.0014 (8)	0.0043 (7)
C10	0.0226 (9)	0.0209 (9)	0.0220 (9)	0.0029 (7)	0.0023 (7)	0.0044 (7)
Cd1	0.03076 (9)	0.02554 (9)	0.02014 (8)	−0.00652 (6)	0.00025 (6)	0.00165 (5)
O1	0.0815 (14)	0.0236 (8)	0.0382 (10)	−0.0203 (8)	0.0143 (9)	−0.0043 (7)
O2	0.0522 (10)	0.0328 (8)	0.0241 (7)	−0.0176 (7)	0.0026 (7)	−0.0013 (6)
O3	0.0426 (8)	0.0255 (7)	0.0178 (7)	−0.0125 (6)	0.0021 (6)	0.0009 (5)
O4	0.0446 (9)	0.0285 (7)	0.0157 (6)	−0.0153 (6)	0.0021 (6)	0.0024 (5)
O5	0.0389 (8)	0.0353 (8)	0.0204 (7)	−0.0119 (7)	0.0006 (6)	0.0027 (6)
O6	0.0338 (8)	0.0252 (7)	0.0232 (7)	−0.0036 (6)	0.0030 (6)	0.0072 (5)
O1W	0.0427 (9)	0.0333 (8)	0.0345 (8)	0.0032 (7)	−0.0016 (7)	−0.0098 (7)
O2W	0.0424 (9)	0.0286 (8)	0.0336 (8)	−0.0016 (7)	0.0005 (7)	−0.0062 (6)
O3W	0.0347 (9)	0.0617 (12)	0.0428 (10)	−0.0130 (8)	0.0006 (7)	−0.0060 (9)

### Geometric parameters (Å, °)

**Table:**

C1—O1	1.237 (3)	C9—C10	1.512 (3)
C1—O2	1.260 (3)	C9—H9A	0.9700
C1—C2	1.520 (3)	C9—H9B	0.9700
C2—O3	1.427 (2)	C10—O5	1.237 (2)
C2—H2A	0.9700	C10—O6	1.263 (2)
C2—H2B	0.9700	Cd1—O2	2.2424 (19)
C3—C4	1.383 (3)	Cd1—O1W	2.2957 (19)
C3—O3	1.375 (2)	Cd1—O5	2.2956 (17)
C3—C8	1.394 (3)	Cd1—O2W	2.316 (2)
C4—C5	1.390 (3)	Cd1—O6^i^	2.3596 (15)
C4—H4	0.9300	Cd1—O3	2.4874 (14)
C5—C6	1.372 (3)	Cd1—O4	2.5285 (16)
C5—H5	0.9300	O6—Cd1^ii^	2.3596 (15)
C6—C7	1.394 (3)	O1W—H11W	0.8397
C6—H6	0.9300	O1W—H12W	0.8402
C7—C8	1.380 (3)	O2W—H21W	0.8383
C7—H7	0.9300	O2W—H22W	0.8414
C8—O4	1.379 (2)	O3W—H31W	0.8417
C9—O4	1.420 (2)	O3W—H32W	0.8470
			
O1—C1—O2	125.4 (2)	O2—Cd1—O5	165.45 (5)
O1—C1—C2	115.13 (19)	O1W—Cd1—O5	87.45 (7)
O2—C1—C2	119.45 (17)	O2—Cd1—O2W	94.24 (7)
O3—C2—C1	109.57 (16)	O1W—Cd1—O2W	163.87 (6)
O3—C2—H2A	109.8	O5—Cd1—O2W	81.78 (7)
C1—C2—H2A	109.8	O2—Cd1—O6^i^	90.47 (5)
O3—C2—H2B	109.8	O1W—Cd1—O6^i^	81.05 (6)
C1—C2—H2B	109.8	O5—Cd1—O6^i^	77.63 (5)
H2A—C2—H2B	108.2	O2W—Cd1—O6^i^	108.07 (5)
C4—C3—O3	125.13 (18)	O2—Cd1—O3	67.33 (5)
C4—C3—C8	120.01 (17)	O1W—Cd1—O3	86.56 (6)
O3—C3—C8	114.86 (16)	O5—Cd1—O3	126.40 (5)
C3—C4—C5	119.38 (19)	O2W—Cd1—O3	90.19 (5)
C3—C4—H4	120.3	O6^i^—Cd1—O3	152.55 (5)
C5—C4—H4	120.3	O2—Cd1—O4	128.28 (5)
C6—C5—C4	120.65 (19)	O1W—Cd1—O4	82.00 (7)
C6—C5—H5	119.7	O5—Cd1—O4	65.31 (5)
C4—C5—H5	119.7	O2W—Cd1—O4	82.60 (6)
C5—C6—C7	120.26 (19)	O6^i^—Cd1—O4	139.69 (5)
C5—C6—H6	119.9	O3—Cd1—O4	61.11 (5)
C7—C6—H6	119.9	C1—O2—Cd1	126.53 (13)
C8—C7—C6	119.37 (19)	C3—O3—C2	118.18 (15)
C8—C7—H7	120.3	C3—O3—Cd1	124.97 (11)
C6—C7—H7	120.3	C2—O3—Cd1	116.84 (11)
O4—C8—C7	124.89 (17)	C8—O4—C9	118.20 (15)
O4—C8—C3	114.77 (16)	C8—O4—Cd1	123.27 (11)
C7—C8—C3	120.33 (17)	C9—O4—Cd1	118.19 (11)
O4—C9—C10	108.73 (15)	C10—O5—Cd1	127.30 (13)
O4—C9—H9A	109.9	C10—O6—Cd1^ii^	107.39 (12)
C10—C9—H9A	109.9	Cd1—O1W—H11W	117.3
O4—C9—H9B	109.9	Cd1—O1W—H12W	128.5
C10—C9—H9B	109.9	H11W—O1W—H12W	107.6
H9A—C9—H9B	108.3	Cd1—O2W—H21W	109.9
O5—C10—O6	124.02 (18)	Cd1—O2W—H22W	105.4
O5—C10—C9	120.46 (16)	H21W—O2W—H22W	107.1
O6—C10—C9	115.50 (17)	H31W—O3W—H32W	106.7
O2—Cd1—O1W	99.05 (8)		
			
O1—C1—C2—O3	−178.37 (19)	O2—Cd1—O3—C2	−3.57 (13)
O2—C1—C2—O3	1.9 (3)	O1W—Cd1—O3—C2	−104.87 (14)
O3—C3—C4—C5	−179.58 (19)	O5—Cd1—O3—C2	170.85 (12)
C8—C3—C4—C5	0.4 (3)	O2W—Cd1—O3—C2	90.94 (14)
C3—C4—C5—C6	−0.1 (3)	O6^i^—Cd1—O3—C2	−41.85 (19)
C4—C5—C6—C7	0.1 (3)	O4—Cd1—O3—C2	172.37 (15)
C5—C6—C7—C8	−0.3 (3)	C7—C8—O4—C9	−0.3 (3)
C6—C7—C8—O4	179.02 (19)	C3—C8—O4—C9	178.21 (17)
C6—C7—C8—C3	0.6 (3)	C7—C8—O4—Cd1	172.86 (15)
C4—C3—C8—O4	−179.20 (18)	C3—C8—O4—Cd1	−8.6 (2)
O3—C3—C8—O4	0.8 (2)	C10—C9—O4—C8	173.41 (16)
C4—C3—C8—C7	−0.6 (3)	C10—C9—O4—Cd1	−0.1 (2)
O3—C3—C8—C7	179.33 (18)	O2—Cd1—O4—C8	13.52 (17)
O4—C9—C10—O5	−0.9 (3)	O1W—Cd1—O4—C8	−81.73 (15)
O4—C9—C10—O6	−179.40 (16)	O5—Cd1—O4—C8	−172.60 (16)
O1—C1—O2—Cd1	174.23 (18)	O2W—Cd1—O4—C8	103.08 (15)
C2—C1—O2—Cd1	−6.0 (3)	O6^i^—Cd1—O4—C8	−147.63 (13)
O1W—Cd1—O2—C1	87.61 (19)	O3—Cd1—O4—C8	8.75 (14)
O5—Cd1—O2—C1	−156.6 (2)	O2—Cd1—O4—C9	−173.33 (13)
O2W—Cd1—O2—C1	−83.22 (19)	O1W—Cd1—O4—C9	91.42 (15)
O6^i^—Cd1—O2—C1	168.62 (19)	O5—Cd1—O4—C9	0.55 (13)
O3—Cd1—O2—C1	5.22 (17)	O2W—Cd1—O4—C9	−83.77 (14)
O4—Cd1—O2—C1	0.7 (2)	O6^i^—Cd1—O4—C9	25.53 (18)
C4—C3—O3—C2	6.8 (3)	O3—Cd1—O4—C9	−178.10 (16)
C8—C3—O3—C2	−173.16 (17)	O6—C10—O5—Cd1	−179.96 (13)
C4—C3—O3—Cd1	−172.35 (15)	C9—C10—O5—Cd1	1.6 (3)
C8—C3—O3—Cd1	7.7 (2)	O2—Cd1—O5—C10	159.4 (2)
C1—C2—O3—C3	−176.97 (17)	O1W—Cd1—O5—C10	−83.56 (17)
C1—C2—O3—Cd1	2.2 (2)	O2W—Cd1—O5—C10	84.40 (17)
O2—Cd1—O3—C3	175.58 (16)	O6^i^—Cd1—O5—C10	−164.95 (18)
O1W—Cd1—O3—C3	74.28 (16)	O3—Cd1—O5—C10	0.3 (2)
O5—Cd1—O3—C3	−10.00 (17)	O4—Cd1—O5—C10	−1.19 (16)
O2W—Cd1—O3—C3	−89.91 (15)	O5—C10—O6—Cd1^ii^	−17.0 (2)
O6^i^—Cd1—O3—C3	137.30 (14)	C9—C10—O6—Cd1^ii^	161.49 (13)
O4—Cd1—O3—C3	−8.48 (14)		

Symmetry codes: (i) −*x*+3/2, *y*+1/2, −*z*+1/2; (ii) −*x*+3/2, *y*−1/2, −*z*+1/2.

### Hydrogen-bond geometry (Å, °)

**Table:**

*D*—H···*A*	*D*—H	H···*A*	*D*···*A*	*D*—H···*A*
O1W—H11W···O3W^i^	0.84	2.11	2.892 (3)	154
O1W—H12W···O1^iii^	0.84	1.87	2.686 (2)	164
O2W—H21W···O6^iv^	0.84	2.06	2.873 (3)	165
O2W—H22W···O3W^v^	0.84	2.03	2.860 (3)	170
O3W—H31W···O6	0.84	2.09	2.887 (3)	157
O3W—H32W···O2^vi^	0.85	1.99	2.835 (2)	176

Symmetry codes: (i) −*x*+3/2, *y*+1/2, −*z*+1/2; (iii) *x*, *y*−1, *z*; (iv) *x*, *y*+1, *z*; (v) −*x*+1/2, *y*+1/2, −*z*+1/2; (vi) *x*−1, *y*−1, *z*.
